# Amplifying Panic and Facilitating Prevention: Multifaceted Effects of Traditional and Social Media Use During the 2015 MERS Crisis in South Korea

**DOI:** 10.1177/1077699019857693

**Published:** 2019-07-26

**Authors:** Mihye Seo

**Affiliations:** 1 Sungkyunkwan University, Seoul, South Korea

**Keywords:** social media, traditional media, MERS, negative emotional responses, preventive behaviors

## Abstract

In the context of the 2015 Middle East respiratory syndrome (MERS) outbreak in South Korea, this study examines the multifaceted effects of media use considering the current complex media environment. Analysis of a two-wave online panel survey found that traditional media use had a positive influence on MERS knowledge while social media use did not. However, knowledge did not facilitate preventive behaviors. In contrast, negative emotional responses due to media use stimulated desirable behaviors. Furthermore, social media use directly influenced behavioral responses but traditional media use did not show the same effects. Different functions of traditional and social media during an epidemic are discussed.

In 2015, the second largest outbreak of Middle East respiratory syndrome (MERS) in the world hit South Korean society hard. Within a 2-month period, a total of 186 cases were confirmed, and 36 people died (World Health Organization, 2015). MERS was thought to be mainly contained in Middle Eastern countries, with rather limited transmission from human to human, but the 2015 outbreak in South Korea (hereafter Korea) changed that understanding considerably (Petersen, Hui, & Zumla, 2015). This unforeseen crisis induced not only morbidity and mortality but also fear and panic in Korea. In fact, the panic epidemic caused more widespread damage than the disease itself by slowing the economy and interfering with people’s daily routines.

In times of crisis, the importance of the media is heightened. Government and responsible organizations consider media to be an essential part of crisis management (Reynolds & Seeger, 2005), and the public relies on the media to make sense of confusing or chaotic situations (Tai & Sun, 2007; Zhang, Kong, & Chang, 2015). Given the importance of media in times of crisis, scholarly attention has been largely paid to the following questions: (a) how government and other organizations work (or should work) with media to prepare for and respond to crises and (b) how the media reports (or should report) on crises. Relatively less attention, however, has been paid in existing research to examining informational, emotional, and behavioral consequences of individuals’ media use in times of crisis.

As the importance of social media has risen in general, its importance in the context of crises has also increased. Evidence shows that many people turn to social media to seek crisis-related information, such as safety instructions and news updates (Veil, Buehner, & Palenchar, 2011), which stands to promote proper behavioral responses to facilitate effective crisis management. However, both researchers and practitioners caution that media—social media in particular—may create misperceptions and amplify public fears by fostering public panic and proliferating unverified information (Kasperson, 1992). In comparison to traditional media, social media use is particularly susceptible to the aforementioned concerns due to enhanced speed of information transmission and distinctive features of open access platforms (Zeng, Starbird, & Spiro, 2016).

In the context of the 2015 MERS outbreak in Korea, this study provides an empirical examination of the multifaceted effects of media use in times of crisis in the complex and dynamic media environment of today. Using two waves of online panel data collected at two different time points during the MERS crisis, I investigate how individuals’ traditional and social media use during the crisis produced various consequences, including increased MERS knowledge, negative emotions such as fear and anxiety, and direct and indirect facilitation of MERS preventive behaviors. I also scrutinize the differences in these effects caused by traditional and social media use.

## Past Research on the Role of Media During Crises

The term *crisis* is defined as “some breakdown in a system that creates shared stress” (Coombs, 2014, p. 2), which includes a very broad range of situations. An infectious disease outbreak is a typical example of crisis in the public health context. Prior research has focused on how governments or other responsible organizations can achieve positive relationships with the public in managing a particular crisis. Based on the organization–public relationship (OPR) approach, scholars have theorized and investigated various (pre)conditions, attributes, and communication strategies of organizations to bring about positive relational outcomes with the public, such as satisfaction, commitment, trust, and mutual understanding (S.-U. Yang, 2018). With respect to the MERS crisis in Korea, S.-U. Yang (2018) showed that the government’s lack of dialogic competency negatively affected government–public relationships. Those findings indicate that the Korean government’s lack of mutuality and openness weakened the credibility of its risk information, which produced negative relational consequences such as distrust and dissatisfaction and the intent to dissolve the relationship.

Cooperation with the media on the part of government and responsible organizations is a major portion of effective crisis management processes (Coombs, 2014). From a crisis management perspective, prior research has mainly focused on how to understand and work with the media to accomplish various goals (Reynolds & Seeger, 2005). For instance, researchers have identified the kinds of communication strategies that work best to reduce public-relations damage and generate compliance with desired behaviors in hazardous situations (Glik, 2007). For instance, Seeger, Reynolds, and Sellnow (2009) emphasized the importance of coordinating specific communication tasks for each crisis phase in the context of Hurricane Katrina and the H5N1 outbreak. Based on the existing literature and case studies, scholars have also attempted to provide guidelines for best practices in crisis communication (Veil et al., 2011), which could also be used as evaluative criteria in crises (Plattala & Vos, 2012).

Another line of research focuses on how media channels cover crises by analyzing the content of crisis reporting and discussing its implications. As manifested by terms such as *disaster marathon* (Liebes, 1998), the unexpected and impending nature of crises triggers media hype, which produces a prolific amount of reporting. Much research has investigated the characteristics of crisis coverage (Shih, Wijaya, & Brossard, 2008). Shih and colleagues (2008), for instance, found that the coverage of epidemics showed common patterns across discrete diseases, such as a high event-base and emphasis on newly identified cases and government actions. With respect to the MERS crisis in Korea, Jin and Chung (2018) performed semantic network analysis of Korean and foreign media coverage of the crisis. They examined the most frequently used words (e.g., patient, hospital, infection, government, and case) and concluded that Korean media focused heavily on the number of cases and the government’s responses, consistent with Shih and colleagues’ findings (see Kwon, 2016, for similar findings). Based on content analyses of crisis reporting, past research has also identified persistent problems in crisis reporting, such as excessiveness (Rezza, Marino, Farchi, & Taranto, 2004), inaccuracy (Auter, Douai, Makady, & West, 2016), and sensationalism (Moeller, 1999). Korean media’s MERS reporting was not exempt from sensational and excessive coverage of the contagious nature of the disease and patient counts (Kim, 2016; Kwon, 2016).

## Public Responses and Media Use During Public Health Crises

As population mobility and trade in goods and services have increased, newly emerging infectious diseases have become global public health concerns. Some emerging infectious diseases have derived from a known infection, such as influenza, and have spread into new populations. The MERS outbreak in Korea can be understood as one such example. An outbreak of infectious disease causes not only human casualties but also massive economic harm. Different from chronic health risks, infectious pandemics trigger spontaneous and intense media attention (Posid, Bruce, Guarnizo, Taylor, & Garza, 2005), which could create cascading effects in various public responses. However, relatively little empirical research has considered the various consequences of media use by individuals during a public health crisis.

One of the most desirable public responses to a public health crisis is engaging in preventive behaviors (Mitroff, 2004). Public adoption of precautionary behaviors is critical to preventing large outbreaks of infectious disease, particularly in densely populated countries such as Korea. People need to behave in ways that prevent the spread of infectious disease and its consequences, and the media plays an important role in facilitating those behaviors (Gammage & Klentrou, 2011; Zhang et al., 2015). Learning from media is one potential pathway to engagement in preventive behaviors (Sayavong, Chompikul, Wongsawass, & Rattanapan, 2015). Besides cognitive responses, another way to galvanize preventive behaviors could be through emotional responses, which could alarm people enough to take proper actions with respect to a given risk. Research on risk and health communication has offered various theoretical models and empirical evidence for each approach (Boer & Seydel, 1996; Griffin, Dunwoody, & Neuwirth, 1999). Yet, there has been little research testing and comparing the two potential paths to preventive behaviors in the context of a pandemic crisis.

### Cognitive and Behavioral Responses

First, media use could increase knowledge about a crisis, which could stimulate the public to enact preventive behaviors. The heavy emphasis on knowledge is largely drawn from the traditional *Knowledge Deficit Model of Communication* (Rutsaert et al., 2014), which claims that a lack of understanding is the major obstacle to reasonable public responses to a risk or crisis. Therefore, it accentuates the scientific knowledge transfer from experts to the layperson and media have been regarded as a major conduit of knowledge transfer (Hilgartner, 1990). Thus, when a crisis happens, government and responsible organizations attempt to work with media to disseminate crisis-related information, and the general public turns mainly to the media to acquire the information to deal with the atmosphere of uncertainty. Despite that widespread expectation, relatively little empirical attention has been given to whether public crisis knowledge is indeed increased by media use or whether understanding of a crisis indeed facilitates preventive behaviors in times of crisis.

Media is known to be more suitable for diffusion of knowledge than other channels, such as interpersonal communication (Price & Oshagan, 1995). Prior research shows that media use increases health knowledge in the general public, which in turn encourages desirable health behaviors (Gammage & Klentrou, 2011; Sayavong et al., 2015). Little empirical evidence, however, has been collected in the context of urgent public health crises such as epidemics. On the contrary, disaster studies have extensively examined individual and group responses to impending threats (e.g., natural disasters or terrorist attacks). According to that body of research, in the face of an impending threat, people become more sensitive to cues about social environments and engage in searches for information as a basis for protective behaviors (Lindell & Perry, 2004). However, despite the known contribution of media channels to these disaster research models, media variables have received insufficient attention in explaining the behavioral responses of individuals to crises.

Second, media use could stimulate proper behavioral responses via mobilizing information (MI). In the health communication literature, MI is designed to encourage a specific health behavior (Friedman & Hoffman-Goetz, 2003). Applied to a crisis context, MI offers specific “how to” and “where to” information, such as checklists for preparedness supplies, evacuation information, phone numbers or websites for further information, or specific instructions for precautionary behaviors, meant to encourage people to take specific actions (Tanner, Friedman, & Barr, 2009). Facing a crisis, the public needs to learn about both the nature of the crisis and how to mitigate its effects and defend themselves (Guion, Scammon, & Borders, 2007).

A handful of prior studies in the communication discipline have documented the direct and indirect effects of media use on preventive behaviors via knowledge in a crisis situation. Ho (2012), for example, found that attention to newspaper and television news increased public knowledge about the H1N1 pandemic. Zhang et al. (2015) also found that media use potentially influenced H1N1 preventive behaviors through fear and perceived knowledge. The results of 36 national surveys in the United Kingdom indicate that exposure to media coverage or advertising about swine flu increased the adoption of recommended preventive behaviors (G. Rubin, Potts, & Michie, 2010). Lin and Lagoe (2013) also showed that TV and newspaper use increased H1N1 risk perception and vaccination intent in media users. Based on those discussions and findings, it is expected that media use will facilitate public understanding of an emerging infectious disease and encourage appropriate precautionary behaviors. Media use in large-scale emergencies, however, still requires empirical scrutiny because of the many unexpected twists that characterize fluid crisis situations.

### Emotional and Behavioral Responses

Human beings facing a crisis often experience a range of negative emotions. The intense uncertainty inherent to a crisis situation galvanizes fear, worry, and panic (Sandman & Lanard, 2003). In the outbreak of an unfamiliar contagious disease, both the unknown cause and fatal outcome and the interruptions of daily routines and stigma could strengthen negative emotional responses (Lee, Kim, & Kang, 2016). Prior research indicates that individuals often feel more threatened during crises than is warranted by the actual risk level (Coombs & Slovic, 1979). According to *Social Amplification Theory*, the risk people feel when facing a crisis could be amplified or weakened by exchanging various forms of information via the news media or informal networks (Renn, Burns, Kasperson et al., 1992). Once a perceived risk is officially acknowledged, the distortion and exaggeration of information tend follow (Song, Song, Seo, Jin, & Kim, 2017), feeding a range of negative emotional responses.

Prior research has found that the media tends to overemphasize risk and sensationalize crises. For instance, the media overstresses the horrific symptoms of contagious diseases regardless of facts about the prevalence of those symptoms in a time of outbreak (Moeller, 1999; Ungar, 2008). The media also tends to focus more on the spread of a disease and the body counts rather than scientific causes (D. Rubin & Hendy, 1977). These types of sensationalism can produce disproportionate public fear and panic responses to infectious diseases. Scholars such as Muzzatti (2005) have gone a step further and demonstrated that the media can actually manufacture threats to public health. Some prior works have examined the effects of media use on emotional reactions toward a crisis. Hoffner, Fujioka, Ibrahim, and Ye (2002), for instance, found that people who learned about the September 11 terrorist attacks through mass media were more likely to report negative emotions than those who heard the news interpersonally. They attributed that difference to the nature of the live pictures and content in mass media crisis reporting.

People’s negative emotional responses as influenced by media use could lead to inappropriate behaviors, such as avoidance of precautionary behaviors or unnecessary or excessive behavioral reactions (Liu, Hammitt, Wang, & Tsou, 2005). However, other convincing literature has claimed that emotions can trigger behavioral responses that are benign and adaptive (Baumeister, Vohs, DeWall, & Zhang, 2007). Negative emotional experiences can stimulate people to seek pertinent information (e.g., the *Risk Information Seeking and Processing* (*RISP*) *Model*, Griffin et al., 1999) and encourage them to take preventive actions (e.g., *Protection Motivation Theory* [*PMT*], Boer & Seydel, 1996). Unlike the cognition-based approach, which emphasizes the role of knowledge, these theoretical models focus on how negative emotions work as motivational drivers to guide people to protect themselves. Empirical work has supported those claims by showing that negative emotion can drive positive behavioral responses, including adopting recommended health behaviors (Ruiter, Abraham, & Kok, 2001) and engaging in information seeking (Z. J. Yang & Kahlor, 2013).

As discussed, Korean media coverage of the MERS crisis did not deviate wildly from the patterns reported in the literature (Kim, 2016; Kwon, 2016). The terms most frequently and centrally mentioned by the major news outlets mainly related to the contagious nature of the disease and patient counting (Kwon, 2016). Sensational reporting and delivering government press releases without critical validation were also characteristic of the MERS coverage (Kim, 2016). In addition, poor government handling of that crisis reduced the credibility of the information it provided (S.-U. Yang, 2018). Against that backdrop, it is worth investigating the potential association between media use related to MERS and the negative emotional responses of media users to determine the extent to which they influenced the behavioral responses of those users.

## Social Media Use and Effects During Crises

Along with traditional media channels such as television and newspapers, the importance of social media increases during large-scale events. These trends are particularly salient in the context of crises, which are traditionally marked by high levels of information seeking by the general population. Evidence shows that people turn to social media during times of crisis to find information about safety instructions, news updates, and damage reports. Increasingly, the public expects even official agents to respond to public requests via social media in times of emergency, concurrent with traditional crisis management (Veil et al., 2011). These heightened expectations are due in large part to perceptions of the benefits of social media for crisis communication.

It is believed that social media can accelerate information dissemination in crisis situations by linking end users directly to critical information sources in real-time (Hughes & Palen, 2012). During the 2009 H1N1 virus outbreak, people exchanged information and experiences through social media (Chew & Eysenbach, 2010). Social media, such as Twitter, has been used to quickly share initial information and updates during various types of crises, as well as to encourage specific actions, such as volunteering and precautionary behaviors (Potts, 2014). The presence of social media as an information source becomes salient when traditional media provides limited information. For instance, Tai and Sun (2007) showed that, during the severe acute respiratory syndrome (SARS) epidemic in 2003, Chinese people turned to the Internet and message services to find information unavailable from the traditional media, which operate under close censorship by the Chinese government. Likewise, at the beginning of the MERS crisis, the Korean government withheld the list of hospitals affected by MERS, and the mainstream media adhered to the information embargo requested by the government. Limited access to needed information fueled fear, so people turned to social media instead of traditional media outlets (Kim, 2016). Social networking service (SNS) users are likely to be exposed to messages that affect their perceptions and their use of preventive behaviors related to health risks and the crisis event. Vos and Buckner (2016) found that SNS messages in the 2013 outbreak of the H7N9 virus consisted mainly of sense-making messages to educate users about the nature of the risk and efficacy messages to encourage appropriate responses. In a similar line, Yoo, Choi, and Park (2016) found that receiving information about MERS through SNS directly encouraged MERS preventive behavioral intentions.

Social media is filled not only with information but also emotional expressions (Chew & Eysenbach, 2010). Negative emotions are more likely than positive emotions to be floating around social media during an infectious outbreak, which could increase risk perception (Choi, Yoo, Noh, & Park, 2017). For instance, Song et al. (2017) found that negative emotions (e.g., anxiety or fear) prevailed in online boards and social media during the MERS crisis. Often, information intertwined with emotional markers travels around social media, and emotionally charged messages are more likely than purely informational posts to be shared and diffused (Pfeffer, Zorbach, & Carley, 2014; Stieglitz & Dang-Xuan, 2013). S. Choi, Lee, Pack, Chang, and Yoon (2016) mined Internet media reports about MERS in 2016 and examined their effects on public emotion expressed online, which they captured using a sentiment analysis. They mined all MERS-related news articles from 153 media companies, including the comments about each one. In a time-series analysis, they found a flow from Internet media to public fear. According to Song et al. (2017), negative emotion accounted for 80% of all posts throughout online networks, especially Twitter, during the MERS crisis.

Therefore, I have drawn the following four hypotheses. I expected that both traditional and social media use in times of crisis could directly and indirectly facilitate preventive behaviors (via MERS knowledge) and negative emotional responses to the MERS situation.

**H1**: (a) MERS-related traditional media use and (b) social media use will be positively associated with MERS knowledge.
**H2**: (a) MERS-related traditional media use and (b) social media use will be positively associated with negative emotional responses about MERS.
**H3**: (a) MERS knowledge and (b) negative emotions about MERS will be positively associated with MERS preventive behaviors.
**H4**: (a) MERS-related traditional media use and (b) social media use will be positively associated with MERS preventive behaviors.

### Comparison Between Social Media and Traditional Media

Emerging social media channels have reshaped the crisis information context, which potentially complicates the relationships among media use and informational, emotional, and behavioral responses in the general public. Although social media has been considered a powerful tool for disseminating information, both researchers and practitioners have cautioned against the propensity of social media to proliferate inaccurate data, unverified rumors, and even malicious misinformation (Zeng et al., 2016). Because information disseminated through social media is often unverified, identifying accurate data and valid sources can be challenging and could both undermine relevant knowledge and exacerbate the consequences of emotional and behavioral responses. Together with the characteristics of open access platforms, this concern is heightened by the dynamic nature of crisis communication and information overload in times of crisis (Zeng et al., 2016).

Social media networks are mostly composed of acquaintances and thus share common characteristics with interpersonal channels. Prior works have shown that interpersonal channels tend to show stronger effects on behavioral change than traditional media, mainly through normative pressure (Price & Oshagan, 1995). In addition, social media generally deal with a rapid exchange of information, which could be more appropriate for short and clear MI than for scientific knowledge about a crisis. For instance, Won, Bae, and Yoo (2015) conducted an issue word analysis of SNS messages during the MERS outbreak and reported the six most frequently mentioned words on SNS at that time. Two of the six words were *mask* and *hand sanitizer*, which are clearly related to preventive behaviors. The other four words were *hospital, infection, coughing*, and *checkup*, which are also at least indirectly related to preventive behaviors. However, a big data analysis examining online news sites found that posts about symptoms, government reaction, disease treatment, business impacts, and rumors ranked higher than prevention-related information (Song et al., 2017). Therefore, the effects of social media use on crisis knowledge and preventive behaviors might differ from those of traditional media.

In terms of emotional responses, social media and traditional media might also show some differences. Social media is known for its ability to spread information in a speedy and viral manner, but that information tends to be integrated with emotions. Furthermore, emotionally charged messages are more likely to be shared than neutral messages (Pfeffer et al., 2014; Stieglitz & Dang-Xuan, 2013). Recent works on the MERS crisis have shown that about 80% of the buzzwords on social media during the MERS crisis were negatively charged emotional words such as fear and anxiety (S. Choi et al., 2017; Song et al., 2017). Those works suggest that social media might be more likely than traditional media to magnify negative emotional responses. On the contrary, some prior works have found that people use social media for social support in crisis situations (Y. Choi & Lin, 2009). That phenomenon could lessen the negative emotional responses in social media users. Therefore, it is worth investigating how the cognitive, emotional, and behavioral responses to social media use differ from those to traditional media use, which raises the following research question:

**RQ1**: Are there any differences between traditional media use and social media use in terms of cognitive, emotional, and behavioral responses during the MERS crisis?

**Figure 1.**
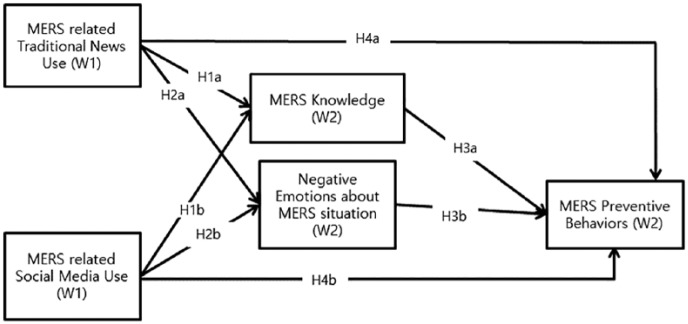
Hypothetical model. *Note*. Variables (W1) = measured in Wave 1 survey; Variables (W2) = measured in Wave 2 survey. MERS = Middle East respiratory syndrome.

The proposed research model is based on the preceding discussion (see Figure 1). Specifically, I expect that both traditional and social media use related to MERS was associated with MERS knowledge, and that it directly and indirectly encouraged MERS preventive behaviors in media users. I also expect that traditional and social media use about the Korean MERS crisis stimulated negative emotional responses, which in turn influenced both precautionary and panic behaviors in media users. Finally, I examine whether social media use showed informational, emotional, and behavioral consequences similar to those of traditional media use.

## Method

### Data Collection and Sample

The data for this study were obtained from a two-wave online panel survey. Wave 1 survey was conducted in the first few days of June 2015, when hype about the MERS situation was escalating, and Wave 2 survey was conducted about 1 month later. The respondents were adult members of an online panel recruited and maintained by a survey company in Seoul, Korea. About 1 million online users compose the company’s panel, and participants were randomly drawn from that list to meet the specified constraints (e.g., areas of residence based on census composition). The selected panel members received a soliciting e-mail from the company and had full liberty to decline participation. The first page of the online survey included an explanation of the study purpose and researcher contact information. Study participants gave informed consent by clicking the start survey button. They were free to stop taking the survey at any time, and they received small monetary incentives (e.g., cash-equivalent points) from the company. A total of 1,187 members of the online panel participated in Wave 1 survey. About 1 month later, the same respondents were recontacted by the survey company to participate in Wave 2 survey. The number of participants who completed both Waves 1 and 2 online surveys was 969, which is the final subject sample used for the analyses herein.

The final sample was 50.7% male, with a mean age of 43.88 years (*SD* = 12.67). With regard to level of education, 50.5% of the sample had some level of college education or a bachelor’s degree, 17.1% had a 2-year college degree, and 20.9% of the sample had some level of high school education or were graduates of high school. The median monthly household income was between 3,500,000 and 4,000,000 won. Of the total sample, 64.4% were married, with an average of 1.7 children (*SD* = .84).

### Measures

#### Traditional news use

Traditional news use of MERS-related content was measured based on media use frequency questions. Specifically, on a 7-point scale (1 = *never* to 7 = *very often*), respondents were asked to measure how much they relied on TV news or TV news websites and newspapers and newspaper websites in the context of the Korean MERS crisis (*r* = .37, *M* = 4.20, *SD* = 1.25).

#### Social media use

Social media use of MERS-related content was measured using three 7-point scales (1 = *never* to 7 = *very often*) in Wave 1 survey. The specific use behaviors were (a) seeking MERS-related news or information using social media, (b) receiving MERS-related news or information from others via social media, and (c) talking about MERS with others via social media (α = .79, *M* = 3.72, *SD* = 1.48).

#### Negative emotional reactions to the MERS situation

Negative emotional reactions to the MERS situation were measured in both Waves 1 and 2 surveys. On a 7-point scale (1 = *not at all* to 7 = *feel strongly*), respondents were asked to report how strongly they felt fear and anxiety about the MERS situation (Wave 1, *r* = .85, *M* = 5.42, *SD* = 1.47; Wave 2, *r* = .85, *M* = 5.08, *SD* = 1.53).

#### MERS knowledge

MERS knowledge was measured using six quiz-type questions in both Waves 1 and 2 surveys. Knowledge items covered MERS symptoms, lethality, maximum latent period, and institutions where a formal diagnosis of MERS could be made during the outbreak. For each question, correct answers were coded as 1, and incorrect answers were coded as 0. Subsequently, the answers to those six questions were combined and used as the MERS knowledge measure (Wave 1, with scores ranging from 0 to 6, *M* = 3.09, *SD* = 1.17; Wave 2, with scores ranging from 0 to 6, *M* = 3.75, *SD* = 1.26).

#### MERS preventive behaviors

Preventive behaviors against MERS were measured in Wave 2 survey using five items. On a 7-point scale (1 = *never* to 7 = *very often*), respondents were asked how often they engaged in behaviors to prevent MERS: wearing a mask when they were out, washing hands frequently, avoiding contact with people with MERS symptoms, avoiding hospitals, and avoiding daily activities such as grocery shopping to avoid crowds (α = .77, *M* = 4.18, *SD* = 1.43).

## Results

Before running the path model to test hypotheses, a confirmatory factor analysis (CFA) was conducted to test validity of the measurement model. Two knowledge measures were excluded from CFA because measures were composed of all dichotomized items. Model modification was not utilized: χ^2^(67) = 301.95, *p* = .00; normed fit index (NFI) = .95; incremental fit index (IFI) = .96; comparative fit index (CFI) = .96; root mean square error approximation (RMSEA) = .06 with 90% confidence interval (CI) = [.05, .07]. Factor loadings ranged from .51 to .98 and two factor loadings were rather low of .56 (one of MERS preventive behaviors) and .51 (newspaper use item). However all AVE (average variance extracted) were higher than .50 and factor loadings were still within acceptable range (Fornell & Larcker, 1981). Therefore, all items remained. Path analysis was conducted to test the hypotheses with latent variables and two observed knowledge variables. The tested model included two control variables from Wave 1 data. Specifically, MERS knowledge and negative emotions about the MERS situation measured in Wave 1 controlled each of the Wave 2 variables accordingly. The research model showed a reasonably good fit according to commonly used criteria (Hu & Bentler, 1999): χ^2^(90) = 359.76, *p* = .00; NFI = .96; IFI = .96; CFI = .96; RMSEA = .056 with 90% CI = [.050, .062].

**Figure 2.**
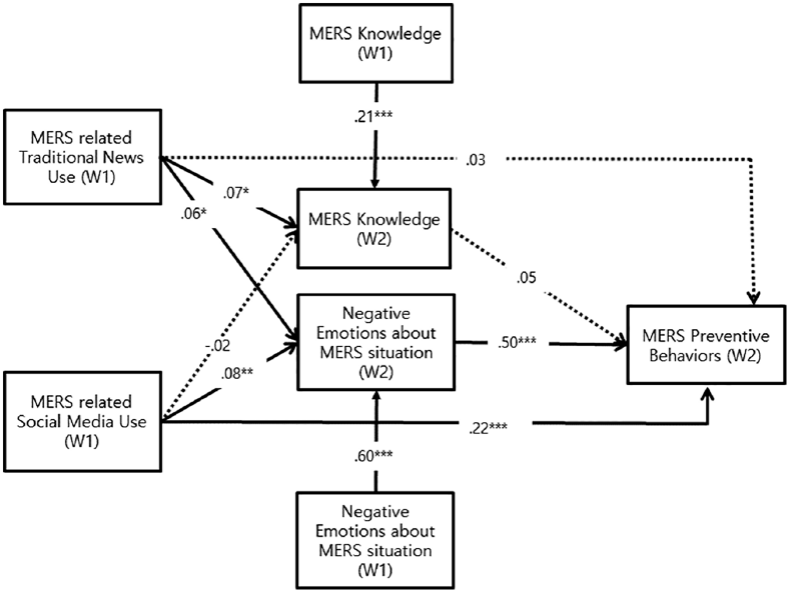
Overall results. *Note*. Dotted arrows denote paths that are not statistically significant or only marginally significant. The numbers presented in Figure 2 are the standardized path coefficients. All exogenous variables, including MERS knowledge (W1) and negative emotions (W1), were correlated with one another. No error term was correlated. W1 = Wave 1; W2 = Wave 2; MERS = Middle East respiratory syndrome. *p < .05. **p < .01. ***p < .001.

Figure 2 presents the overall results with path coefficients of the hypothesized model. **H1** predicted that the more individuals use traditional news media (**H1a**) and social media (**H1b**), the more MERS knowledge they would acquire. As expected, MERS knowledge increased with increasing use of MERS-related traditional news media (β = .13, *p < .*01), which supported **H1a**. On the contrary, MERS-related social media use was not significantly associated with MERS knowledge (β = −.04, *ns*), which did not support **H1b**. Therefore, **H1** was only partially supported. **H2** predicted that two types of media use would be positively associated with negative emotional responses to the MERS situation. Results showed that the more traditional media one used, the more anxious and worried about MERS one became (β = .09, *p < .*05), which supported **H2a**. However, MERS-related SNS use and negative emotions was not significantly associated (β = .05, *ns*), which failed to support **H2b**. Therefore, **H2** was also partially supported.

The next set of hypotheses predicted that positive effects of MERS knowledge (**H3a**) and negative emotional responses (**H3b**) on MERS preventive behaviors. As predicted, negative emotional response was positively related to engagement in MERS preventive behaviors (β = .54, *p < .*001). On the contrary, MERS knowledge was not significantly associated with MERS preventive behaviors (β = .05, *ns*). Therefore, **H3** was also partially supported. The final set of hypotheses expected that both traditional news media use (**H4a**) and social media use (**H4b**) would have direct positive effects on MERS preventive behaviors. Results showed that only social media use showed direct positive effects on MERS preventive behaviors (β = .23, *p < .*001) not traditional media use (β = .05, *ns*).

Finally, **RQ1** asked whether traditional media use and social media use show any differences in terms of cognitive, emotional, and behavioral responses. Results showed that individuals during the MERS crisis showed different responses depending on two types of media use. With respect to cognitive responses, as reported above, only traditional media use was positively associated with MERS knowledge. Likewise, in terms of emotional responses, traditional news media use showed a positive association with negative emotional reactions. With respect to behavioral responses, only social media use showed significant positive direct effects on MERS preventive behaviors. Wald tests comparing the path coefficients confirmed that the difference between effects of social media and traditional media on behavioral responses (Wald test = −6.51, *p* < .001) was statistically meaningful. Wald test result also validated that the difference in cognitive response between social and traditional media use was also statistically significant (Wald test = −2.29, *p* < .01).

## Discussion

Despite a consensus that the media play an important role in times of crisis, the informational, emotional, and behavioral effects of traditional and social media use by individuals have been understudied in previous research. Using two sets of data collected at two different time points during the 2015 MERS crisis in Korea, I investigated how traditional and social media use influenced MERS knowledge, fear and anxiety about the MERS situation, and adoption of preventive behaviors. To reflect the complexity of today’s media environment in times of crisis, I focused on potential differences between the effects of traditional and social media use.

First, this study found that traditional media use and social media use have different effects. As expected, traditional media use galvanized public understanding of MERS. The more people read newspapers and watched TV news about MERS, the more knowledge they acquired about MERS symptoms, lethality, and maximum latent period. Social media use neither increased nor decreased MERS knowledge. The role of social media in times of crisis has received growing attention, at least in part because of its potential for viral information transmission. However, I did not find significant cognitive effects from social media use. Compared with traditional media, which mainly report information verified by expert sources (Lowrey, 2004), social media can not only convey knowledge but also disseminate false or unverified information during a crisis. The null effect of social media use on MERS knowledge might thus result from the conflicting content of social media. This speculation, of course, should be verified with a robust empirical examination.

Second, I found that negative emotions played a prominent role in facilitating preventive behaviors in the MERS crisis. Experiencing fear and anxiety is a natural reaction when people are faced with a crisis. A lack of familiarity with newly emerging infectious diseases such as MERS tends to deepen fear and anxiety (Sandman & Lanard, 2003), and media use could amplify those emotional experiences (Kasperson, 1992). Finding of this study also indicate that traditional media use increased negative emotional responses. The more media respondents consumed, the more fear and anxiety they felt about the MERS situation. More importantly, I found that the negative emotional responses people experienced when facing a crisis caused them to protect themselves from MERS instead of pushing them into illogical or destructive behaviors.

The positive role of negative emotions in promoting adaptive behaviors is consistent with what theoretical frameworks such as PMT and RISP suggest. However, most prior studies based on PMT have been developed and tested within the context of public health campaigns. Compared with carefully designed campaign messages intended to scare people to act properly with respect to a given risk, media messages could more likely be crude and mixed with various intentions (e.g., sensational reports to secure audience attention). Indeed, there is a lack of empirical research examining what kinds of crisis-specific responses could be elicited from the media individuals choose to use in times of crisis. In addition, RISP research provides a useful theoretical framework to predict that negative emotions could motivate people to seek information about a risk. However, this framework did not pay attention to media use, which can lead to behavioral consequences such as preventive behaviors beyond information seeking. This study addressed these gaps in the literature by directly testing the roles of negative emotions and media as potential sources of those emotions during a pandemic, which has been rarely examined in previous studies.

This finding has clear implications for governmental communication strategies and media reporting. This study’s results suggest that it may not be effective crisis management to brush off negative emotions among the public as illogical overreactions. Instead, admitting fears and anxiety people may feel and designing messages channeling those emotions into desirable health behaviors needs to be considered even in times of crisis. Sympathizing with people’s feelings can be an effective means to boost the evaluation of communicators’ dialogic competency which was found to be positively related to information credibility and trust in the government during the MERS endemic (S.-U. Yang, 2018), which in turn may promote government and media health message effectiveness. This seemingly positive role of negative emotions, however, does not advocate for sensationalizing crisis situations. Instead, both government and media may consider applying knowledge about the potentially positive function of negative emotion to public communication during a crisis. For instance, risk and health communication research has developed guidelines on how to write a good fear appeal message in the public health campaign context. Government and news media may consult with these guidelines to compose a message to help people to cope with an epidemic risk.

Third, the findings of this study indicate that the role of knowledge for preventive behaviors in the 2015 MERS crisis was rather limited. Only traditional media use significantly increased the public’s knowledge about MERS but the increased understanding did not facilitate precautionary behaviors. Disseminating knowledge has been a top priority in times of crisis under the assumption that understanding could lead the public to protective behavioral responses. The results of this study imply that filling the deficit of knowledge may not be the most efficient way to promote behavioral responses. The findings here suggest that an emotional pathway may work more efficiently than cognitive drives in promoting the adoption of preventive behaviors during a public health crisis.

However, it is also important to point out that the findings related to knowledge should be interpreted with caution in the context of the 2015 MERS crisis. S.-U. Yang (2018) noted that Koreans in general tended to perceive the government’s mutuality and openness in communication somewhat unfavorably, which often negatively affected government-public relationships. As S.-U. Yang (2018) showed, the Korean government’s lack of mutuality and openness seems to have deteriorated the credibility of its risk information, which may have worsened the public’s trust and satisfaction with the government. This unique situation might have lowered not only the credibility of government information but also the credibility of information from news media that relied heavily on the government for information. Accordingly, the low credibility of public information on MERS can explain the limited role of knowledge in promoting desirable behavioral responses. Prior work examining a different epidemic crisis found that one material factor influencing preventive action was respondent opinion about authorities (Quah & Hin-Peng, 2004). My finding thus suggests that simple acquisition of disseminated scientific knowledge might not necessarily produce desirable behavioral responses from the public. It also implies that contextual factors, such as government dialogic competency, should be considered to fully grasp the role of knowledge in times of crisis.

Fourth, the findings of this study show that, unlike traditional media use, social media use produced strong direct behavioral responses during the MERS crisis. Traditional media use did not show direct effects on preventive behaviors. This distinction between social media use and traditional media use could be explained in the following two ways. First, compared with traditional media, which is known to be powerful in knowledge diffusion, the interpersonal channel has been considered effective on behavioral responses via the normative route (Price & Oshagan, 1995). The role of normative pressure on behavioral adaptation has been well documented in the health communication literature (e.g., *Planned Behavioral Intention Model*, Ajzen, 1991). Given that social media networks are mainly composed of acquaintances, it is possible that social media might have formed specific behavioral norms related to the MERS crisis (e.g., avoidance of hospitals and crowded places).

The other explanation is related to potential differences in the content of traditional media and social media. Social media might have delivered more MI than traditional media. Indeed, a prior work analyzing social media content during the MERS crisis reported that preventive behavior–related terms (e.g., masks and hand sanitizers) were the most frequently mentioned words (Won et al., 2015). Furthermore, the most needed information at the beginning of the MERS crisis was the list of MERS-affected hospitals, which the Korean government had withheld. Given the government’s unwillingness to share the list of hospitals, the public resorted to social media to actively seek, exchange, and share the hospital-related information. That piece of information could single-handedly trigger one preventive behavior (i.e., avoiding specific hospitals to protect themselves). Indeed, *hospital* was one of the terms most frequently used on social media during the MERS crisis (Won et al., 2015). However, a big data analysis of traditional media reported that words such as *infection, confirmed cases*, and *death* were centrally located in traditional media content (Kwon, 2016). In short, compared with traditional media, social media might play an essential role in virally diffusing MI (specific behavioral information, including where not to go and what to do).

Given the importance of dialogic competency for effective crisis communication (S.-U. Yang, 2018), some technological features of social media may have enhanced communication mutuality and openness which the Korean government and media lacked. Communication through social media may allow users to share empathy and social support. At the same time, social media-mediated communication can be perceived as more accessible and open than media-mediated communication. Strong mutuality and openness perceived through communication with network members on social media could have increased the credibility of MI which could have enhanced behavioral responses at least during the MERS crisis in Korea.

Another notable finding regarding social media is that social media did not generate public anxiety and fear, at least during the MERS crisis in Korea. This is quite an interesting finding because the Korean government strongly criticized media, especially social media, for releasing unverified rumors and fears. The lack of association between social media use and negative emotional responses could be a result of canceling out effects. In other words, social media might induce fear and anxiety, but social media might effectively cancel out the negative emotions by providing wanted social supports or MI, which should be under solid empirical scrutiny in the future study.

In the interest of future research, it is important to discuss some of the limitations of this study. The data are not based on a representative sample, which means the study findings must be interpreted with caution. For instance, online panels tended to include the younger and the more educated compared with the general population, which could influence the interpretation of the role of social media and traditional media use during the MERS crisis in Korea. The measurement of key variables also has limitations. For example, traditional media use was assessed only for newspaper and television. Omitting radio and general Internet use should be recognized as another major limitation of the study. Also, using quiz-type questions to tap MERS knowledge without close validation and planning might have contributed to the low correlation between the two MERS knowledge measures, which also raises a concern about a test–retest error involved in the panel data. These measurement issues could produce errors, which would be carried into the hypothesized path model this study proposed. In addition, Wave 1 data did not include MERS preventive behavior measures, which made it impossible to control all the endogenous variables in Wave 1 data. Therefore, it is hard to argue that an ideal panel data analysis was conducted for testing the proposed hypotheses.

With these limitations in mind, this study contributed to the scholarship by testing the predictions drawn from existing theories that have rarely been examined in the context of a real epidemic crisis. Major scholarly focus in the communication field has been on how government and responsible organizations manage or should manage crises. Shifting away from that focus, this study investigated the associations between individual media use and consequent responses which have been relatively understudied.
